# Diagnosis and treatment of Langerhans Cell Histiocytosis with bone lesion in pediatric patient: A case report

**DOI:** 10.1016/j.amsu.2019.07.030

**Published:** 2019-08-02

**Authors:** Achmad Fauzi Kamal, Andi Praja Wira Yudha Luthfi

**Affiliations:** Department of Orthopaedic and Traumatology, Dr.Cipto Mangunkusumo General Hospital/Faculty of Medicine Universitas Indonesia, Jakarta, Indonesia

**Keywords:** Langerhans cell hystiocytosis, Bone lesion, Case report

## Abstract

**Background:**

Langerhans cell histiocytosis (LCH) is a rare group of disorders without a well understood etiology. Known formerly as histiocytosis X, the disease has a wide spectrum of clinical presentations, including eosinophilic granuloma (solitary bone lesion), diabetes insipidus, and exophthalmos. Many of these patients initially present to orthopaedic surgeons, and misdiagnosis is frequent.

**Methods:**

We deliver a case of a 10-month-old boy who consulted to our department. Previously misdiagnosed as a Kawasaki syndrome, TORCH, and osteomyelitis. He had undergone several examinations and had been discussed in clinocipathological conference (CPC) to narrow down the diagnosis.

**Result:**

After serial examinations, the diagnosis of Langerhans Cell Histiocytosis was confirmed and chemotherapy was initiated. And after 6 cycles of chemotherapy, with 1-week interval of each therapy, the clinical appearance of this patient significantly improved.

**Conclusion:**

Despite major advances in our understanding and management of LCH, it remains one of the most challenging diagnoses for the orthopedic surgeon. By doing a comprehensive examination, it is possible to narrowing down the diagnosis and planning the accurate treatment.

## Highlights

• Langerhans cell histiocytosis (LCH), also known as histiocytosis X, is a rare disorder resulting from an abnormal clonal proliferation of macrophages.
• Despite major advances in our understanding and management of LCH, it remains one of the most challenging diagnoses.
• In diagnosing LCH a thorough physical examination, Laboratory studies, and radiological studies are mandatory.
• Many different treatments for multisystem LCH, from minimal therapy to intensive chemotherapy, have been tried.
• Vinblastine and etoposide, have been identified as effective single agents. Both have activity against diverse disorders of the monocyte-macrophage system and combination chemotherapy including them had promising results in multisystem LCH.

## 1 Introduction

Histiocytic disorders constitute heterogeneous group of diseases characterized by accumulation of reactive or neoplastic histiocytes in various tissues. The histiocytic disorders cover a wide range of primary and secondary, solitary and multiple, benign and malignant disorder. Langerhans cell histiocytosis (LCH) is a reactive disorder in which cells having the phenotypic markers of epidermal Langerhans cells are found in skin and other organs where they cause damage by excessive production of cytokines and prostaglandins. Langerhans cell histiocytosis (LCH), also known as histiocytosis X, is a rare disorder resulting from an abnormal clonal proliferation of macrophages. LCH is named as such due to its similarity in both morphology and immunophenotype to Langerhans cells, which are phagocytic cells located in the skin and mucosa. LCH has a heterogeneous clinical presentation involving different organs of the body, such as the bone, skin, lymph nodes, liver, spleen, lung, central nervous system, thyroid, and thymus. Depending on the site of accumulation and proliferation of the clonal cells, the disease can be classified into 3 main categories: unifocal (also known as eosinophilic granuloma), unisystem multifocal, or multisystem [1,2].

In diagnosing LCH a thorough physical examination is mandatory. The skin and mucosal membranes should be inspected, and special attention should be paid to the neurologic status of patients with known or suspected cranial and/or spinal involvement. Laboratory studies should include a complete blood count with differential and smear; complete chemistry profiles, including total protein, albumin, bilirubin, liver enzymes, and alkaline phosphatase; inflammatory markers (erythrocyte sedimentation rate and C-reactive protein level); coagulation studies; thyroid panel; and urinalysis. Radiography of the chest and a skeletal survey are also recommended. Whole-body bone scintigraphy (WBBS) with technetium-99 m is not diagnostic for LCH but may help to narrow the differential diagnosis. WBBS may reveal additional polyostotic lesions in asymptomatic regions that might otherwise not undergo imaging. Finally, WBBS may play a role in monitoring various therapeutic responses [3].

Many different treatments for multisystem LCH, from minimal therapy to intensive chemotherapy, have been tried. Two drugs, vinblastine and etoposide, have been identified as effective single agents. Both have activity against diverse disorders of the monocyte-macrophage system and combination chemotherapy including them had promising results in multisystem LCH. However, most reported studies were retrospective, small, or included patients with highly variable degrees of involvement. Thus, identification of the optimal treatment for multisystem LCH remained unresolved [4]. In this study we will present a case about diagnosis and treatment of LCH and this work has been reported in line with the Surgical Case Report (SCARE) criteria [5]. Patient's parents had been given proper information about this paper and the possibility of publication.

## 2 Methods

A 10-month-old boy with history of intermittent fever since he was 3-month-old, accompanied with hematoschezia, hemoptoe, and irritating. He was brought to previous hospital and told to have a throat inflammation and given antibiotics and vitamins. He was also previously diagnosed with Kawasaki Syndrome, but without convincing examinations. Cytomegalovirus (CMV) infection came up as one of the diagnosis after he had undergone another examination with increasing level of IgG anti-CMV. The doctor gave anti-viral treatment but after 1 week, the fever is still not subsided. His parents refuse any similar signs and symptoms happened to any member of the family before.

**Fig. 1:**
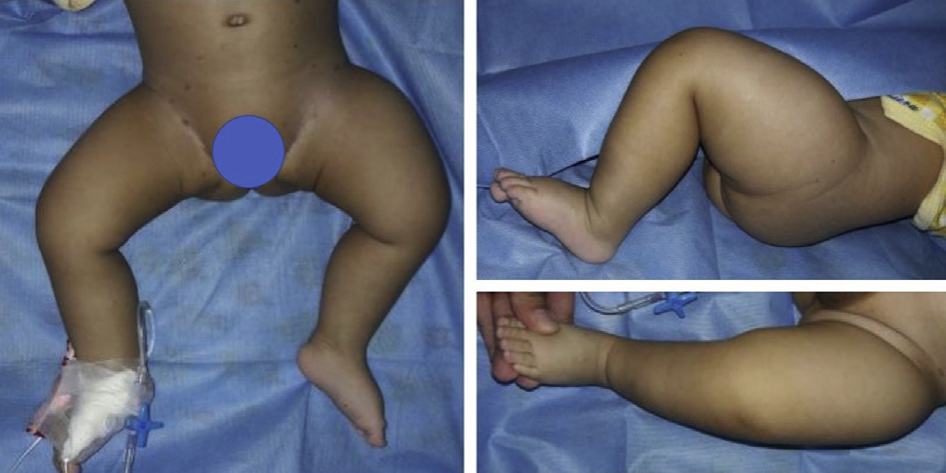
Clinical appearance of left thigh when he was consulted to our department, we could see swelling on left thigh.

The patient was consulted to our department with swollen and painful thigh region (Fig. 1). Plain x-ray, ultrasonography (USG), and Magnetic Resonance Imaging (MRI) were performed. MRI was performed twice in different hospitals for gaining the second opinion. After several examinations, the biopsy was performed by the author to establish the diagnosis. This case had been delivered in clinicopathological (CPC) meeting to established the final diagnosis and designed an accurate treatment plan for this patient. He had already undergone chemotherapy with Vinblastine 1mg and Etoposide 25mg for 12 cycles. The first 6 cycles was therapeutic dose, with 1-week interval for each cycles and last 6 cycles was maintenance dose, with 1-month interval for each cycles.

## 3 Result

**Fig. 2:**
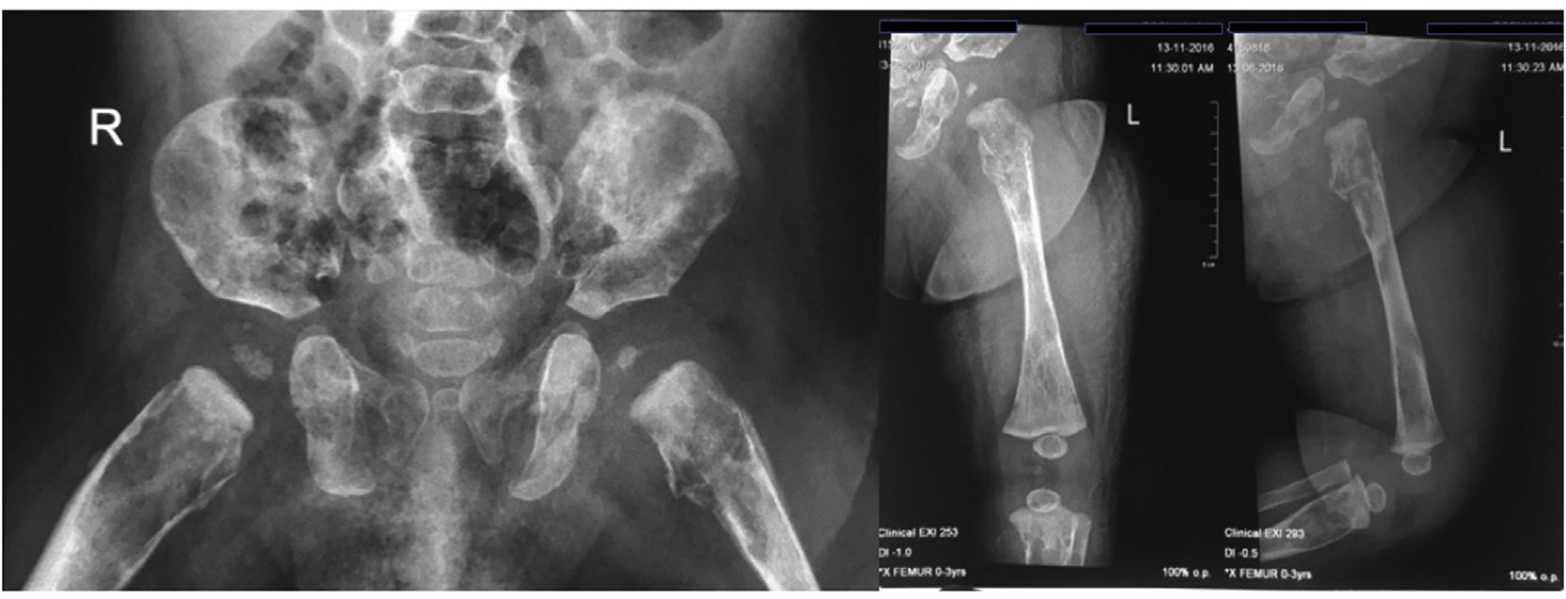
Initial X-rays of pelvic and left femur showed multiple lytic lesions and thinning of bone cortices.

**Fig. 3:**
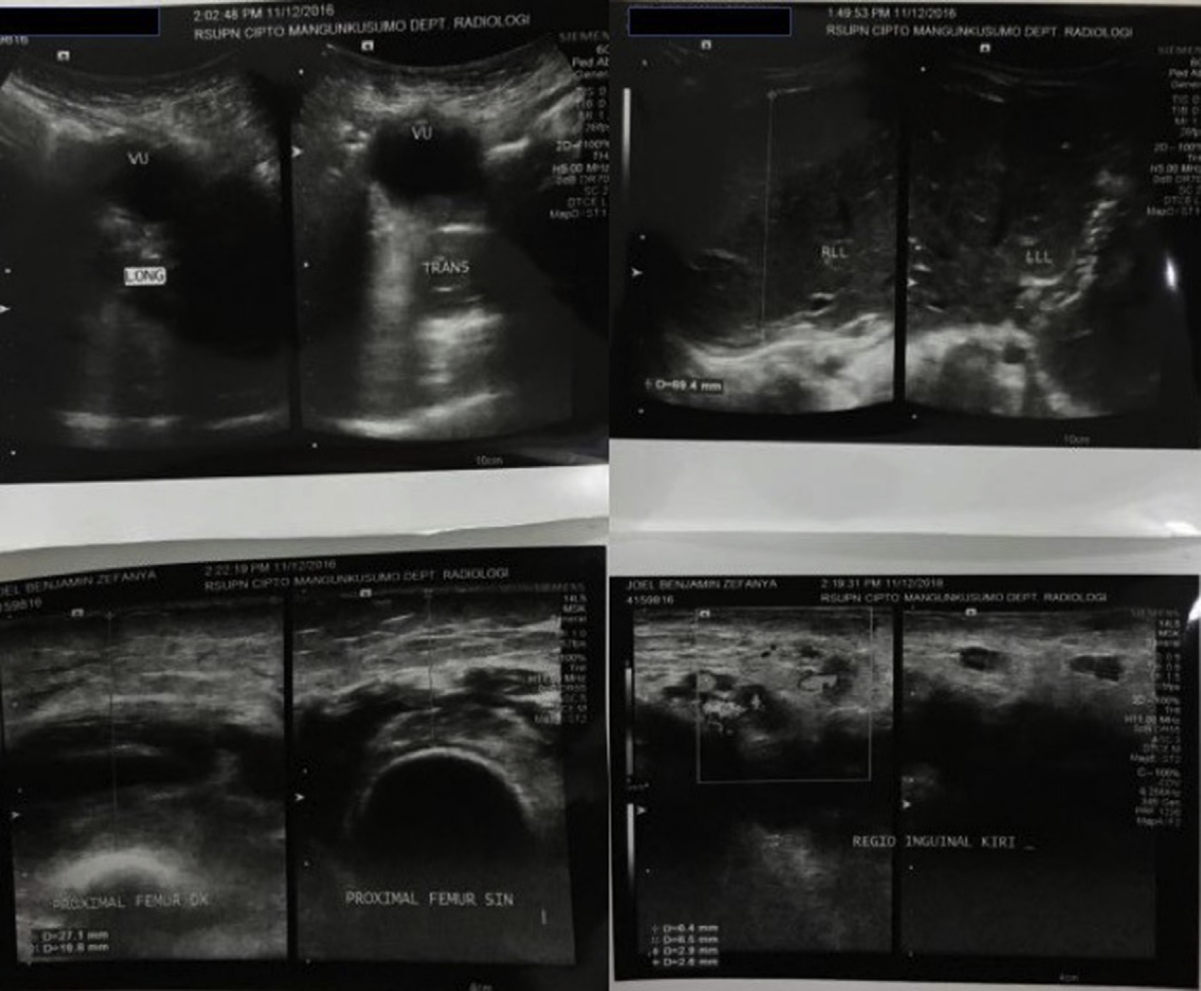
Abdominal USG at left groin area revealed enlargement of lymph nodes (2016).

**Fig. 4:**
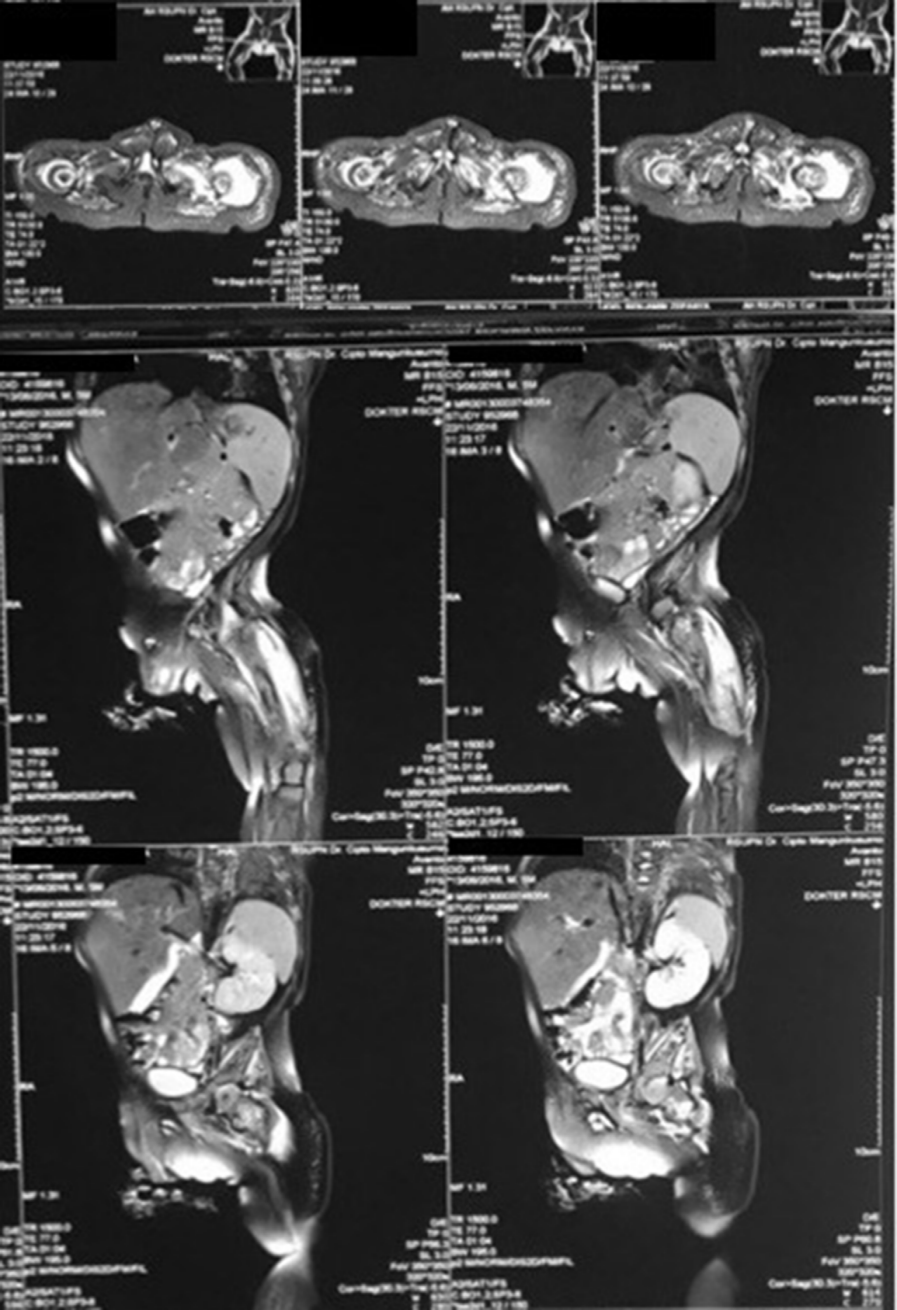
First MRI of pelvic and left thigh concluded multifocal osteomyelitis (2016).

**Fig. 5:**
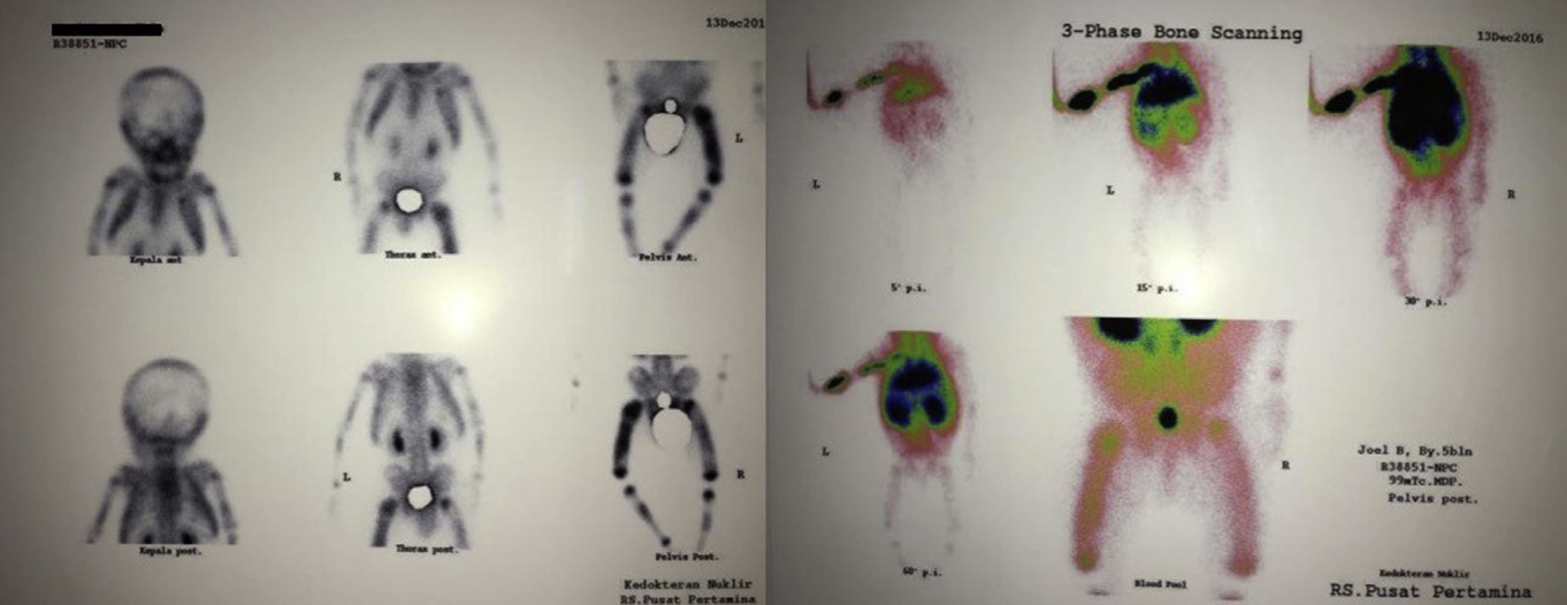
Bone scan examination supported the first MRI result.

**Fig. 6:**
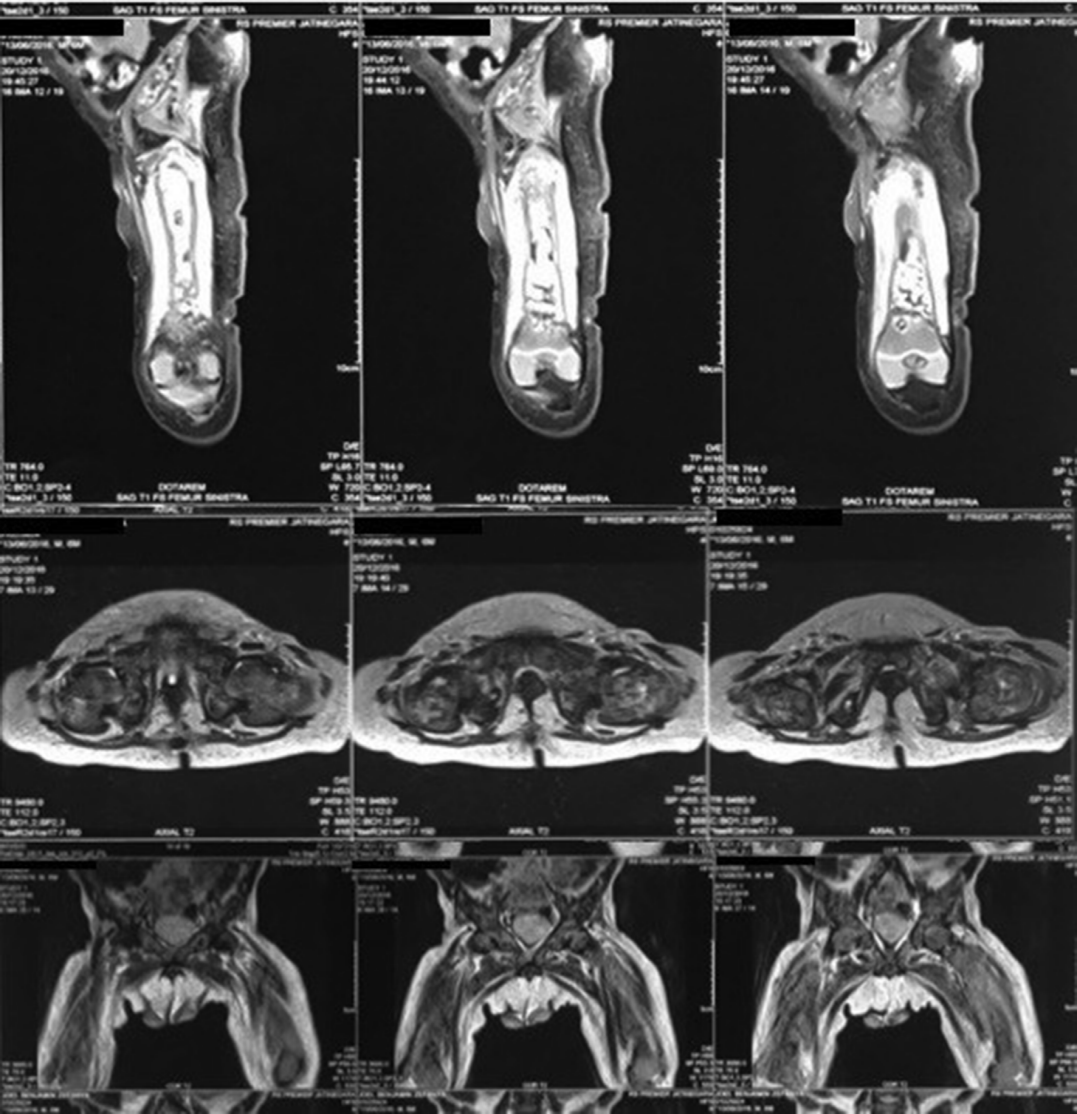
Second MRI was taken to confirm and compare with previous MRI, and the result suggested metastatic neuroblastoma as differential diagnosis.

**Fig. 7:**
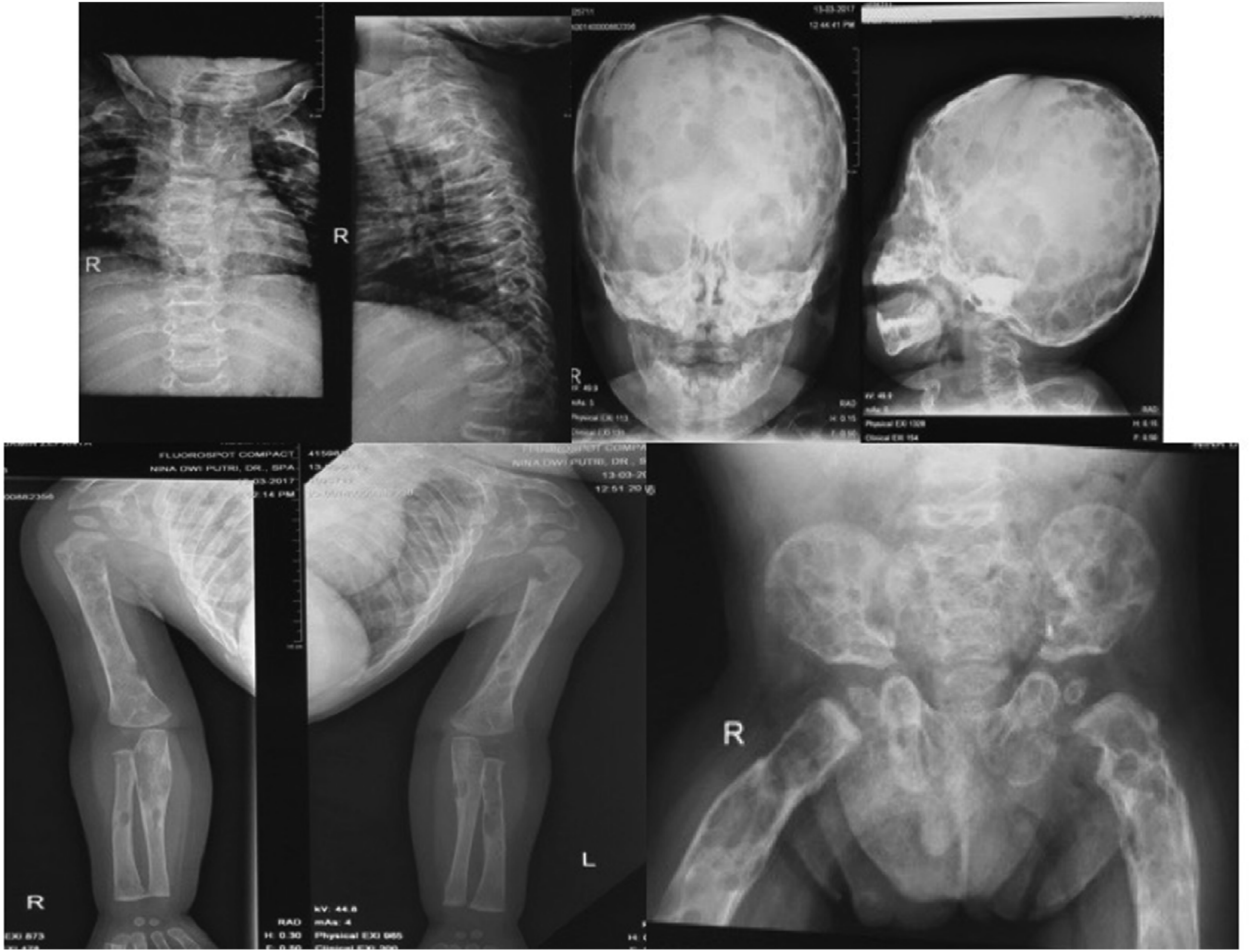
Bone survey revealed multiple osteolytic lesions in the bones. Punch out lesion on the skull was also demonsrated.

**Fig. 8:**
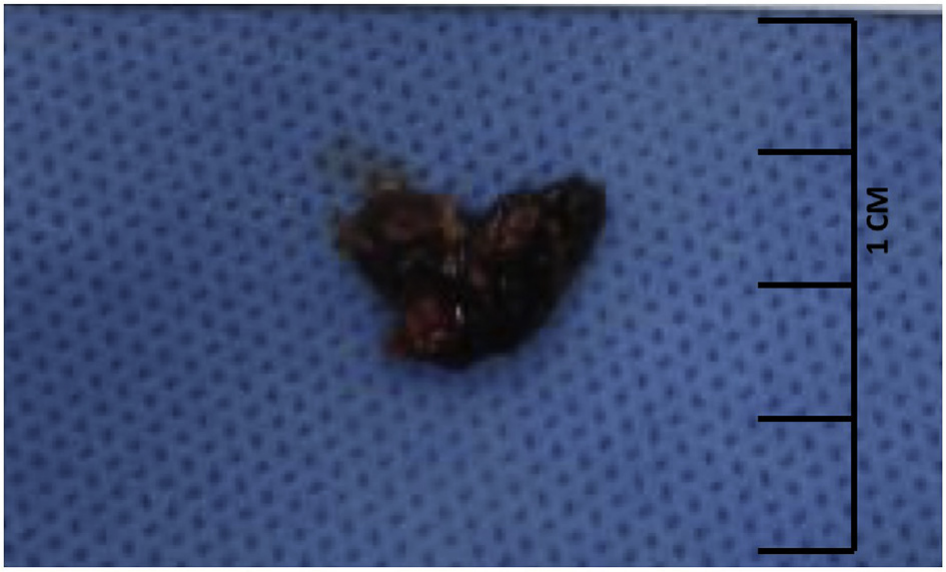
Gross pathology taken by open biopsy of the femur.

**Fig. 9:**
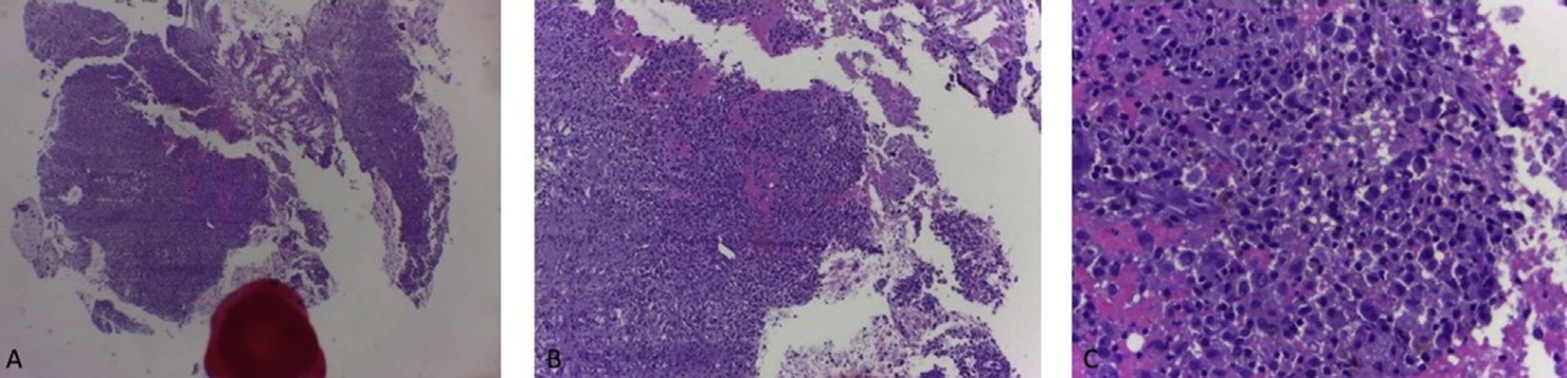
Histological features on 40x (A), 100x (B), 400x (C) magnitude showing oval and round cell nucleus, hyperchromatic and eosinophilic cytoplasm, with Langerhans Cell Histiocytosis as a conclusion.

**Fig. 10:**
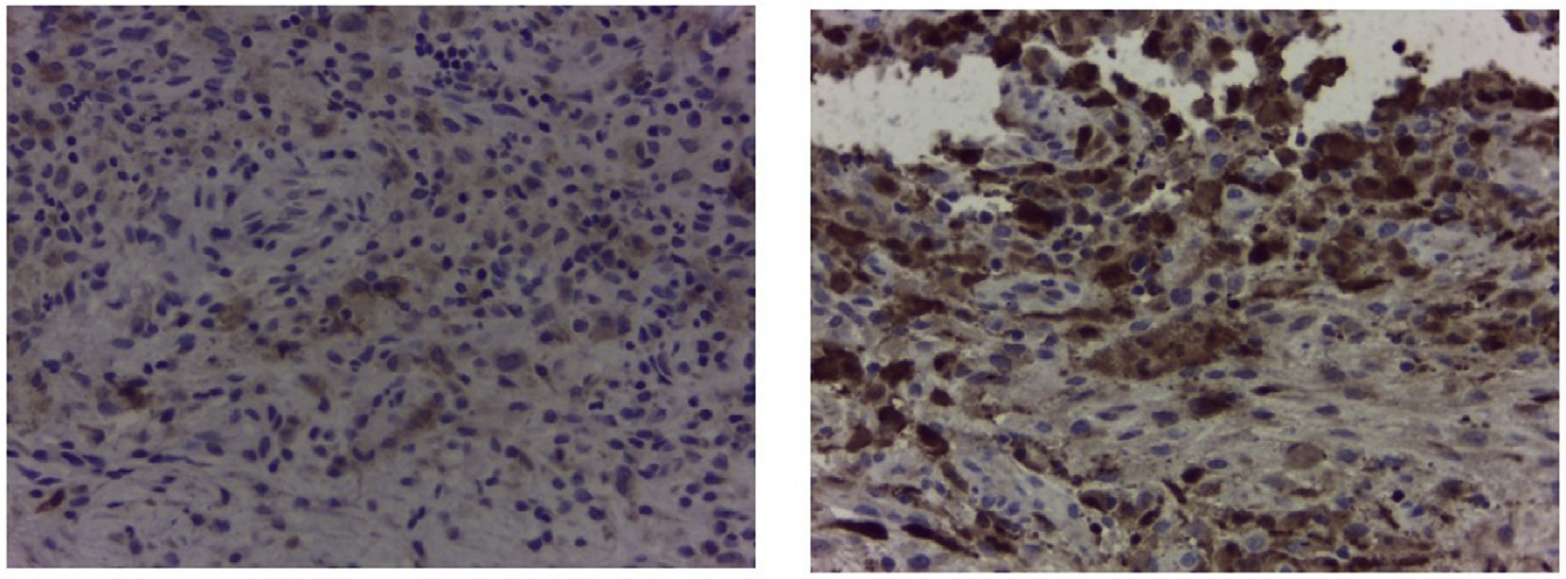
Immunohistochemical examination confirmed histological features.

From the plain X-ray of the femur, there was thinning of the cortex on the proximal part of the femur (Fig. 2). From USG examination, it was revealed that there was enlargement of lymph nodes at the groin area and thickening of the soft tissues around (Fig. 3). From the first MRI, osteomyelitis of left femur came up as the suspected diagnosis, and suggested to have a bone scan examination. From the result of bone scan examination, the diagnosis of osteomyelitis was also suspected (Fig. 4, Fig. 5). From the second MRI, the result revealed that the lesion on his femur might be from tumor metastases (Fig. 6). Bone survey examinations was done with the result of generalized moth-eaten destruction with suspected of LCH and lymphoma (Fig. 7), and from the biopsy result, LCH was confirmed, supported by immunohistochemical examination (Fig. 8, Fig. 9, Fig. 10**)**.

After the chemotherapy, significant improvement in patient's signs and symptoms was showed. He was more active, no fever, no sign of allergy reaction, and the patient gained more weight.

## 4 Discussion

Langerhans cell histiocytosis (LCH) represents a spectrum of rare disorders characterized by idiopathic infiltration and accumulation of abnormal histiocytes (i.e. the Langerhans cells) within various tissues (bone marrow, skin, central nervous system, lung, liver, spleen, lymph nodes) causing focal or systemic effects. LCH predominates in children and its annual incidence is estimated at 4.6 per million in children under 14 years of age. LCH was formerly known as “histiocytosis X″, a term that grouped three major syndromes, which are now considered as clinical variants of the same disease: the eosinophilic granuloma (unifocal LCH with a solitary bone lesion), the Hand-Schüller-Christian disease (multifocal LCH with the classic triad of skull lesions, exophtalmos, and diabetes insipidus) and the Letterer-Siwe disease (fulminant LCH with multiple organ involvement). The aetiology of LCH remains un-known, and it is still uncertain whether LCH is a neoplastic disorder, suggested by the monoclonality in lesions, or a reactive disorder resulting from a dysregulation of the immune system. Nowadays, this old terminology has been replaced by a classification system that is based on the site of lesions, number of involved sites (single or multisystem/local or multifocal), and whether the disease involves risk organs (hematopoietic system, liver, or spleen) [6,7]. Letterer-Siwe disease is seen in infants (usually within the first year of life), but occurrence in adult has been reported. These patients present with fever, weight loss, pancytopenia, lymphadenopathy, hepatosplenomegaly, cutaneous lesions, and bone lesions. Hand-Schüller-Christian disease is seen in younger children who present with osteolytic skull lesions, diabetes insipidus (due to hypopituitarism), and exophthalmos. Eosinophilic granuloma develops primarily in older children and adults who present with solitary bone lesions, skin lesions, or lymphadenopathy. CSR is seen at birth or in the neonatal period with only cutaneous lesions [8,9].

Although major advances have increased our understanding of LCH, its pathophysiology remains unknown and may be the result of either an aberrant autoimmune response or a neoplastic process. A viral or other infectious agent has been considered because of the self-limiting nature of the disease in some patients and their response to antibiotics and steroids. However, no organism has been isolated from lesions of LCH. There are insufficient data to support metabolic and genetic factors or immunodeficiency as causes. Evidence in support of LCH as runaway immunogenic response to an external inflammatory stimulus includes the fact that lesions have extensive inflammatory characteristics and often spontaneously resolve. The issue of a viral triggering agent in LCH has been raised again, some years after others have found no evidence of many other viruses, by Murakami et al., who recently reported elevated amounts of Merkel cell polyomavirus DNA in the peripheral blood cells in 2 of 3 LCH patients with high-risk organ involvement and lower levels in an additional 12 LCH tissues. This finding is interesting but deserves confirmation in other series. The possible correlation of alterations described in LCH, such as the novel cell fusion pathway in an ex vivo model, the excess of spontaneous chromosomal breaks recalling the genomic instability induced by respiratory syncytial virus, hepatitis C virus or Epstein-Barr virus. However, the clonal nature of Langerhans cells within LCH lesions and the presence of the recently discovered BRAF gene mutations (a proto-oncogene that encodes the signaling of cell growth) in more than 50% of LCH cells are highly suggestive of a neoplastic process. At this time, neither environmental factors nor infectious agents have been clearly associated with the disease. Significant debate continues about the true nature of LCH lesions [3,10,11].

Clinical presentations of LCH vary widely, from an asymptomatic solitary bone lesion to a multisystem life-threatening affliction (Table 1**)**. Bone is the most commonly affected system; bone lesions are present in approximately 80% of patients with LCH. The most common site of involvement is the skull (27%), followed by the femur (13%), mandible (11%), and pelvis (10%). Mostly, radiographic studies typically show lytic lesions, especially punched-out lesions in the skull without marginal sclerosis or periosteal reaction. Pain and tumor formation in a localized area of bone is a very common presentation of LCH. In the skull, the lesions are usually soft and tender to touch. Skull lesions may include a soft tissue mass pressing on the dura, but severe intracranial extension is rare. Involvement of the skull base is also very common in LCH; typical locations include the bones of the orbit or the temporal bone (typically the mastoid). Patients with LCH lesions of the spine frequently present with localized neck and/or back pain followed by restricted spine motion. Pain caused by these lesions is substantially more common in adults than in children. Up to 50% of monostotic LCH cases are thought to occur in the spine. Spinal involvement and neurologic symptoms are more common in adults than in children and may include corresponding radicular nerve pain, muscle weak-ness, and bowel and bladder dysfunction [3,6,10]. In our case, the patient was irritating and pain on his lower limbs. The pain also accompanied with swelling that made the previous doctor thinks towards infection.

**Table 1:** Clinical manifestation of extraskeletal LCH.

Symptom	Causes	Recommended Clinical Tests
Thirst, polyuria	Diabetes insipidus (pituitary involvement)	Head MRI, urine and plasma osmolality, water deprivation testing
Decreased energy, weight gain, lethargy, cold intolerance	Hypothyroidism (thyroid or hypopituitary axial involvement)	TSH, free T4, head MRI
Lethargy, pallor, history of bleeding disorders, tachycardia	Pancytopenia, anemia, (marrow infiltration, associated malignancy)	Anemia studies, marrow aspirate
Enlarged lymph nodes	Lymph node involvement	Brain, CT-CAP or PET-CT
Cough, dyspnea, tobacco use	Pulmonary involvement	Smoking cessation (if applicable), chest radiograph and CT-CAP pulmonary function tests
Purpuric rashes/mucosal lesions	Skin involvement	Skin biopsy
Diarrhea, weight loss, malabsorption symptoms of hematoschezia	Gastrointestinal involvement	Endoscopy with biopsy or capsule endoscopy, stool studies
Hearing impairment, chronic otorrhea	Mastoid involvement	Head MRI, formal hearing assessment

Symptoms, Causes, and Recommended Clinical Tests for Common Extraskeletal Manifestations of Langerhans Cell Hystiocytosis.

CT-CAP = CT of chest, abdomen, and pelvis with oral and intravenous contrast, PET-CT = positron emission tomography CT, TSH = thyroid stimulating hormone, T4 = thyroxine.

Laboratory studies should include a complete blood count with differential and smear; complete chemistry profiles, including total protein, albumin, bilirubin, liver enzymes, and alkaline phosphatase; inflammatory markers (erythrocyte sedimentation rate and C-reactive protein level); coagulation studies; thyroid panel; and urinalysis. European guidelines also support the use of abdominal ultrasounds, especially in young children, to evaluate for LCH lesions of the liver, spleen, and abdominal lymph nodes. Imaging plays a crucial role in the diagnosis and management of LCH, especially in the case of bone lesions. Prior to confirming a diagnosis of an isolated bone lesion of LCH, a skeletal survey should be performed to screen for other lesions. The course of this disease is unpredictable due to the possibility of progression or spontaneous resolution of the lesion. Therefore, routine imaging must be done throughout the course of the selected management modality and long after its resolution to rule out recurrence. Once a bone lesion is diagnosed, computed tomography (CT) or magnetic resonance imaging (MRI) may be necessary to assess precisely the degree of trabecular and cortical bone destruction in areas at risk of impending fracture and to guide a bone biopsy if necessary (CT), or to assess the degree of soft tissue infiltration in areas at risk of neurological complications (MRI). Bone scintigraphy may be a complementary technique to the radiographic skeletal survey in assessment of bone involvement, but its sensitivity is limited as well, especially in the skull, and this modality has radiation issues. Recently, new imaging techniques, such as positron emission tomography-computed tomography (PET-CT) and whole-body MRI, have developed in view of an improved assessment of the extent and severity of the disease. Positron emission tomography-computed tomography (PET-CT) provides in-formation related to disease activity and response to therapy, but the inherent radiation burden is questionable. Whole-body MRI can detect extra-skeletal and skeletal lesions without use of ionizing radiation, but its exact role in the diagnostic algorithm of LCH needs further investigation and access to this technique is still limited [6,7,10,11].

From the bone survey examination in our case, we found that our patient had multiple lytic lesions from the skull, humerus, forearm, spine, pelvis, and femur. Previously misdiagnosed as osteomyelitis because the lesion didn't involve the epiphysis area, which supported by bone scan result. MRI was done twice to confirm the diagnosis, first result was osteomyelitis, and in second MRI the result was metastatic neuroblastoma as differential diagnosis. These inconsistencies of the results may be because the LCH is mimicking other diseases that the doctors are familiar to.

To establish the diagnosis of LCH, a tissue biopsy for histologic evaluation is required. For patients with multisystem or multifocal single-system bone disease, core needle or open biopsy of the most suitable lesion should be performed. Fine needle aspiration is inadequate. For low-risk patients with unifocal single-system bone lesions, the overall benefits of biopsy are less certain. Others strongly advocate for needle or open biopsy in almost all circumstances. In LCH, the proliferating cells have characteristic cytologic features of Langerhans Cell. They are ovoid or elliptic in shape with grooved, folded, indented, or lobulated nuclei having inconspicuous nucleoli. Cells in mitosis are variable in number depending on the case, but atypical mitosis is primarily absent. The cells have fairly abundant, weakly eosinophilic cytoplasm. Associated features in the background include increase in eosinophils, neutrophils, and monocyte-derived histiocytes. Plasma cells are virtually absent in many instances [8,11]. Immunohistochemical staining is the process by which proteins can be identified in tissues that are stained using specific fluorescently tagged antibodies. Positive staining for CD1a and/or CD207 proteins (also known as Langerin) is required for the definitive diagnosis of LCH. CD1a and CD207 are transmembrane proteins expressed in LCH lesions and normal dermal Langerhans cells. These proteins are thought to play a role in antigen presentation. From open biopsy examination in our case, we found that the cells were LCH, and confirmed by immunohistochemical examination.

There are no clear guidelines for the treatment of LCH, primarily due to its rarity and variability in location, type, and severity. LCH presenting as a multifocal or multisystem disease most often requires more aggressive treatment, such as chemotherapy. Children with multisystem LCH benefit from treatment regimens with cytotoxic drugs or corticosteroids, alone or in combination. Radiation and chemotherapy have been used for unifocal lesions that fail spontaneous resolution and are difficult to resect surgically. Such lesions could cause permanent complications if left untreated, such as diabetes insipidus, orthopedic problems, facial asymmetry, residual proptosis, and loss of dentition. However, chemotherapy is primarily used for multifocal and multisystem lesions [2,[12], [13], [14]].

The most commonly used therapy for multifocal skeletal LCH consists of steroids and vinblastine (VBL), a relatively non-toxic, and well tolerated combination. Front line treatment of LCH is based on the association of VBL 6 mg/m^2^ i.v. weekly bolus for 6 weeks, with prednisone 40 mg/m^2^/day given orally in three divided doses for 4 weeks and then tapered over the following 2 weeks. After the first 6 weeks of treatment, disease status should be reevaluated and treatment continued accordingly. The evaluation of the disease response is usually classified as ‘‘better’’ in case of complete resolution or regression of the disease, ‘‘worse’’ in case of progression of the disease, and ‘‘intermediate,’’ in case of stable or mixed response with new lesions in one site, and regression in another site. Other evaluation methods have been proposed such as the disease activity score. On the other hand, Etoposide has been used for the treatment of malignant and reactive histiocytoses. In LCH it has been given intravenously mainly to children at a dose of 100–150 mg/m^2^ in combination with other cytotoxic agents or, more recently, as monochemotherapy in which it is proving effective [[14], [15], [16]]. our patient recently given combination of Vinblastine 1mg and Etoposide 25mg per cycle, and already done 6 cycles from 12 cycles planned. From clinical appearance, we evaluate an improvement in activity where the irritating sign was gone and already gained 2kg of weight since the firs chemotherapy.

This study revealed that LCH is still easily misdiagnosed and will lead to under-treatment of the patients. The limitation of this study is we only deliver 1 case of LCH that previously misdiagnosed, when we believe that there are still many misdiagnoses of LCH cases out there especially in developing country like Indonesia. Further sophisticated study should be performed to establish a proper protocol among the patients and clinician in diagnosing the LCH case.

## 5 Conclusion

Despite major advances in our understanding and management of LCH, the disease remains no less strange with its diverse symptoms, variable organ involvement, and mysterious etiology (neoplastic process or autoimmune disease), LCH remains one of the most challenging diagnoses for the orthopedic surgeon. Thus, the surgeon should consider LCH in the differential diagnosis of the child who presents with a radiolucent lesion. This may shorten the time to definitive diagnosis, avoid unnecessary diagnostic and/or surgical procedures, and ensure systemic evaluation and initiation of appropriate care. Our results suggest that combination of VBL and Etoposide are therapeutic option for treatment in multifocal LCH, and support the continued evaluation of VBL and Etoposide in prospective trials.

## Conflicts of interest

The authors declare that there is no conflict of interests regarding the publication of this paper.

## Patient consent

Patient's parents had been given proper information about this paper and the possibility of publication.

## Consent

Written informed consent was obtained from the patient for publication of this case report and accompanying images. A copy of the written consent is available for review by the Editor-in-Chief of this journal on request.

## Statement of ethics

The authors have no ethical conflicts to disclose.

## Provenance and peer review

Not commissioned, externally peer reviewed.
